# Cryoablation and immunotherapy: an overview of evidence on its synergy

**DOI:** 10.1186/s13244-019-0727-5

**Published:** 2019-05-20

**Authors:** B. M. Aarts, E. G. Klompenhouwer, S. L. Rice, F. Imani, T. Baetens, A. Bex, S. Horenblas, M. Kok, J. B. A. G. Haanen, R. G. H. Beets-Tan, F. M. Gómez

**Affiliations:** 1 Department of Radiology, The Netherlands Cancer Institute, Plesmanlaan 121, Amsterdam, 1066 CX The Netherlands; 2 GROW School for Oncology and Developmental Biology, Maastricht University Medical Center, P.O. Box 5800, 6202 AZ Maastricht, The Netherlands; 3 Department of Radiology, Interventional Radiology Service, Memorial Sloan Kettering Cancer Center, 1275 York Ave, New York, USA; 4 Department of Urology, The Netherlands Cancer Institute, Plesmanlaan 121, 1066 CX Amsterdam, The Netherlands; 5 Department of Oncology, The Netherlands Cancer Institute, Plesmanlaan 121, 1066 CX Amsterdam, The Netherlands; 6 Department of Interventional Radiology, Hospital Clinic Universitari, Carrer de Villarroel 170, 08036 Barcelona, Spain

**Keywords:** Cryoablation, Immunotherapy, Cancer, Immune checkpoint inhibitor

## Abstract

Cancer cells can escape the immune system by different mechanisms. The evasion of cancer cells from immune surveillance is prevented by immune checkpoint inhibitors, allowing the patient’s own immune system to attack their cancer. Immune checkpoint inhibitors have shown improvement in overall survival for melanoma, lung cancer and renal cell carcinoma in clinical trials. Unfortunately, not all patients respond to this therapy.

In cancer management, percutaneous ablation techniques are well established for both cure and local control of many tumour types. Cryoablation of the tumour tissue results in cell destruction by freezing. Contrary to heat-based ablative modalities, cryoablation induces tumour cell death by osmosis and necrosis. It is hypothesised that with necrosis, the intracellular contents of the cancer cells stay intact allowing the immune system to induce an immune-specific reaction. This immune-specific reaction can, in theory, also affect cancer cells outside the ablated tissue, known as the abscopal effect. Unfortunately, this effect is rarely observed, but when cryoablation is combined with immunotherapy, the effect of both therapies may be enhanced. Although several preclinical studies demonstrated a synergistic effect between cryoablation and immunotherapy, prospective clinical trials are needed to prove this clinical benefit for patients. In this review, we will outline the current evidence for the combination of cryoablation with immunotherapy to treat cancer.

## Key points

- It is hypothesised that cell death by cryoablation leaves the intracellular contents of the cancer cells intact for the immune system to induce an immune-specific reaction also known as the abscopal effect.
- Immunotherapy uses the immune system for treatment of the tumour, but not all patients respond to immunotherapy.
- Combination of cryoablation with immunotherapy may enhance the effect of both therapies for better tumour destruction.

## Introduction

**Fig. 1:**
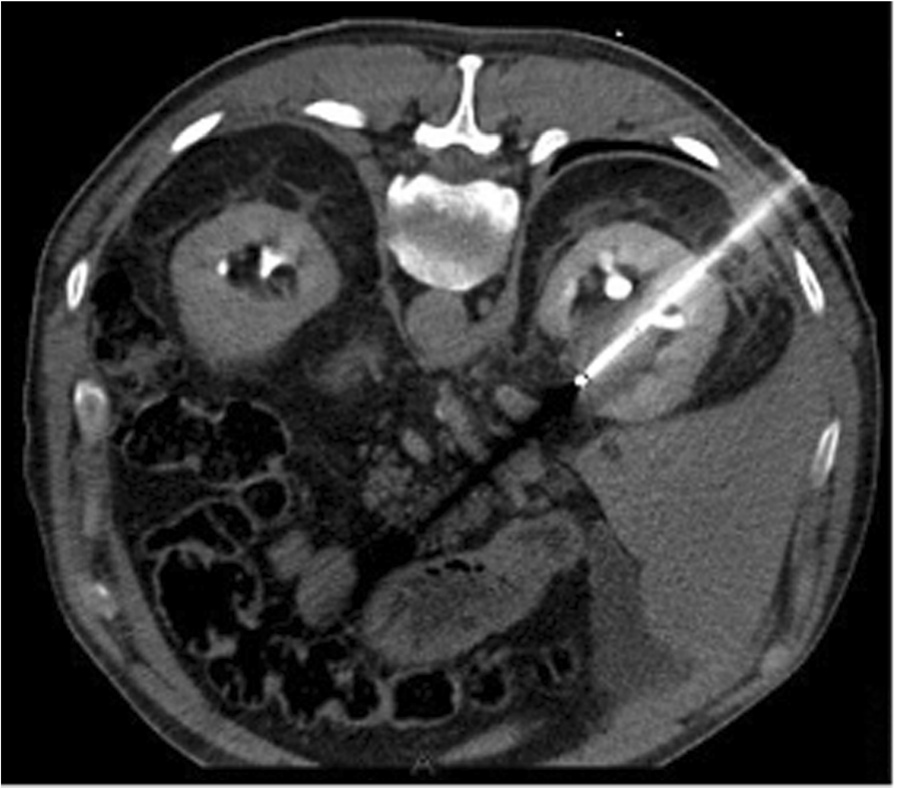
Cryoablation of a patient with stage IA renal cell carcinoma. The hypodense area around the needle corresponds with the generated ice ball in real time

**Fig. 2:**
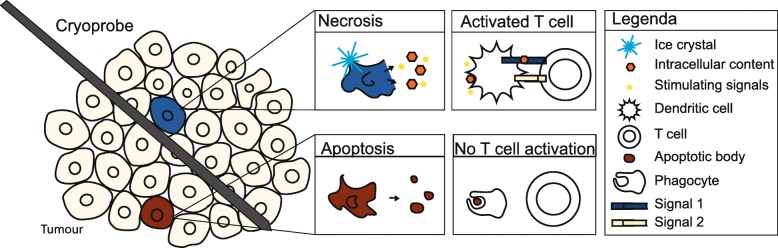
One of the hypotheses of how cryoablation induces an immune response is in the way cell death is induced. Cryoablation induces cell death by both necrosis and apoptosis. Necrosis releases intra-cellular contents stimulating signals (among others danger signals) that may activate T cells for a specific immune response to the cryoablated tissue. Contrary, after cell death by apoptosis, only apoptotic bodies are released, without stimulating signals. Without these stimulating signals, T cells are not being activated. Therefore, apoptosis may lead to an immune-suppressing signal

**Fig. 3:**
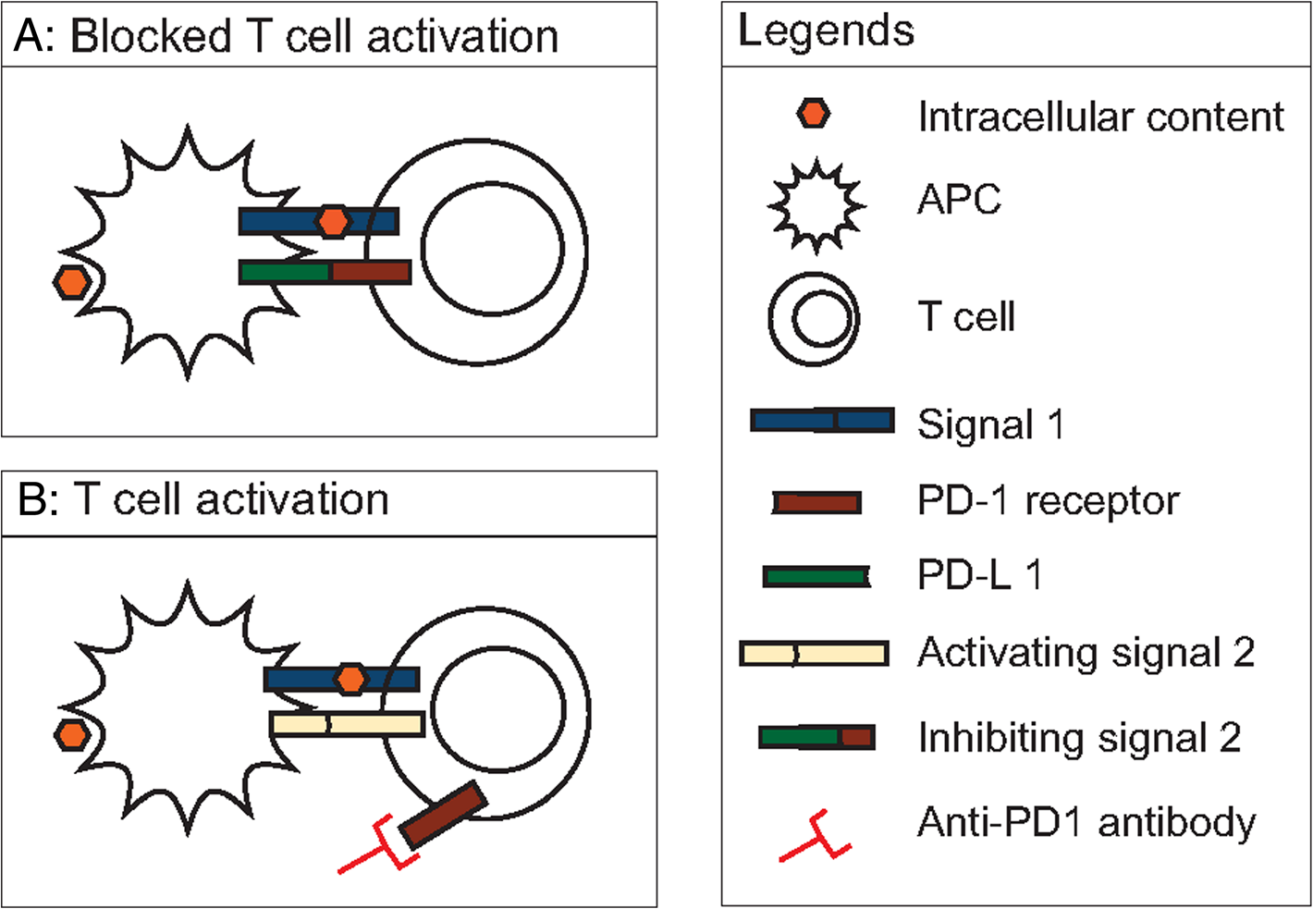
For T cell activation, both stimulatory signal 1 and 2 are needed. Signal 1 consists of the interaction between the major histocompatibility complex on the dendritic cell and the T cell receptor. Different combinations of interactions are possible for signal 2. One inhibiting signal 2 is the interaction between programmed death receptor 1 (PD-1) on T cell and programmed death ligand 1 (PD-L1) on tumour cells or antigen presenting cells. **a** T cell activation is blocked by an inhibiting signal 2 between PD-L1 and PD-1 receptor binding. **b** When an anti-PD1 antibody is used, the inhibiting signal of the T cell is blocked whereby an activation signal of the T cell is gathered and the T cell is activated

Cryoablation is a percutaneous ablation technique that uses extreme low temperatures for tumour destruction [1]. During cryoablation liquefied gas, such as nitrogen or argon, is passed through cryoprobes and expands into a gaseous state at the end of the probe to create temperatures as low as − 190 °C. Cytotoxic cell destruction is achieved at temperatures below − 20 °C. To ensure complete ablation of the tumour, a circumferential margin of 1 cm is needed [2]. After the freezing phase, a thawing phase follows by replacing the liquefied gas with helium or internally heating the needle (available in new systems). The whole process of freezing-thawing is repeated to obtain an effective ablation. Intra-procedure computed tomography (CT) identifies the ablated zone in real time as a low-density area which corresponds to the generated ice ball (Fig. 1). The procedure results in focal destruction of tumour tissue in a minimal invasive setting with reduced morbidity and mortality and represents a cost-effective alternative to surgery [3, 4]. However, surgery remains the gold standard in most tumour types with additional pathological assessment of margins [5, 6]. A possible advantage of cryoablation is that the intracellular contents of the damaged tumour cells are preserved and can be recognised by the immune system initiating a tumour-specific immune response (Fig. 2) [7, 8]. Recently, immune checkpoint inhibitors have been studied for treatment of melanoma, renal cell carcinoma (RCC) and non-small lung cancer (NSLC). The challenge of the immune checkpoint inhibitors is that only a minority of patients respond. Therefore, a combination treatment of cryoablation and immunotherapy, might be beneficial to enhance the effect of the immune checkpoint inhibitors (Fig. 3).

In the early 1970s, Shulman et al. reported the production of antibodies specifically against cryoablated tissue in rabbits [9, 10]. This immunogenic effect potentially resulted in a “bystander effect” with regression of tumours outside the primary ablation zone, known as the abscopal effect, firstly defined by Mole after radiotherapy [11, 12]. Unfortunately, this effect occurs infrequently, but when cryoablation is combined with other immunomodulatory therapy, this effect might be enhanced (Fig. 3) [13]. Here, we review the current evidence on the combination of cryoablation with immunomodulatory drugs (immunotherapy).

## Material and method

A literature search in MEDLINE was performed for available publications between 2001 and 2017 about cryoablation, its effect on the immune system and the combination of cryoablation and immunotherapy. Only full text articles available in English were included. Keywords included cryoablation, immune system, immunotherapy, melanoma and cancer. Data collection included factors related to the synergy between cryoablation and the immune system, cryoablation and immunotherapy, effect of cryoablation on tumour tissue and cryoablation technique. For immunotherapy, all therapies which could theoretically enhance the anti-cancer immune response through either stimulation of the adaptive or innate immune system were included. Studies could be performed in patients and animals. Articles about combination of different ablation modalities were excluded.

A total of 45 relevant papers were identified and the most interesting and relevant studies were used for this review. To outline this paper, the studies were structured by tumour type and the current knowledge about the tumour type, cryoablation and immunotherapy was reviewed.

## Results

### Cryoablation and the immune system

Cryoablation may be synergistic with the immune system in the way cell death is induced. After ablation, the tumour remains, releasing various factors attracting the immune system. A proposed theory regarding the potential mechanisms of cryoablation and the immune system is the danger theory of Matzinger [14, 15]. This theory proposes that after cell death by necrosis, cells secrete danger signals. These danger signals can initiate an immune response. In addition, these signals can mature dendritic cells (DCs) to fully activate T cells which may lead to a specific immune response. Cryoablation induces cell death by necrosis in which intracellular contents are still preserved while DNA, RNA and heat shock protein (HSP), which can induce danger signals, are released [16]. On the other hand, the cells in the outer margin of cryoablated tissue die from apoptosis and do not release DNA, RNA and HSP, and with no danger signal, the DCs remain immature. Immature DCs may trigger immune suppressive signals that could lead to anergy (T cell inactivation) [17, 18]. Therefore, cryoablation can induce both an immunostimulatory and immunosuppressive response (Fig. 2).

In addition, cytokines are produced after cryoablation, and these can also influence an immune response. Again, both immunosuppressive and immunostimulatory cytokines may be released depending on the tumour tissue, age and freeze rate [19]. For cryoablation of liver tumours, when more than 20% of the liver volume is ablated, a systemic inflammatory response can occur due to a release of cytokines interleukine-6 (IL-6), IL-10 and tumour necrosis factor alpha (TNFα), which can have marked systemic effects [7, 20–22]. Therefore, cryoablation is not the preferred treatment since heat-based ablations are better established in this setting [23]. Yet, two clinical studies reported favourable outcomes on overall survival when cryoablation was combined with immunotherapy of allogenic natural killer (NK) cell infusion and dendritic cell cytokine-induced killer (DC-CIK) cells [24, 25].

Preclinical work from den Brok et al. confirms the effect of cryoablation on the immune system. This study reported that cryoablation creates an antigen depot resulting in maturation of DCs. The maturation of DCs led to a tumour-specific immune response protecting half of the mice against a new injection of similar tumour cells. When cryoablation was combined with an immune checkpoint inhibitor, anti-cytotoxic T lymphocyte-associated protein 4 (CTLA-4) antibody, this anti-tumour effect was further enhanced with up to 80% of the mice becoming tumour free [26].

Clinical work by Takaki et al. evaluated peripheral blood after heath- and cold-based ablation modalities with pre-treatment (baseline) in different tumours showing no change in T cell subtypes (regulatory T cell (Tregs), T1 helper or Th2 helper cells) in all modalities, an elevation of cytotoxic T cells after heat-based ablative treatment was noted, and this was not identified after cryoablation [27]. In a group of hepatitis B-positive hepatocellular carcinoma patients, the presence of elevated programmed cell death protein 1 (PD-1) on T cells and programmed cell death ligand 1 (PD-L1) expression on tumour cells had a poor overall survival post cryoablation [28]. Theoretically, when combining cryoablation with a PD-1 inhibitor, such as nivolumab or pembrolizumab, the cryoablation induced adaptive immune resistance with upregulation of PD-L1 on tumour cells could be overcome, resulting in an effective anti-tumour T cell response. This potential synergy between cryoablation and anti-PD-1 may result in a more effective disease control; see Fig. 3 [29].

To enhance the immunogenicity effect of the cryoablation, different immunotherapies can be given as shown in Table 1. Most immunotherapy enhances the innate immunity, namely natural killer (NK) cell therapy, dendritic cells (DCs) and CpG oligonucleotide (CpG ODN). When cryoablation presents the contents of the tumour cells, the innate immune system can assimilate these contents and present it to T cells; an immune response specific to the tumour cells could be obtained. Table 2 is an overview exhibiting the most studied factors after cryoablation in the reviewed papers.

**Table 1:** Summary of the major therapies with their mode of action used in combination with cryoablation in the reviewed papers. All therapies stimulate the immune system in a way and in combination with cryoablation an enhancement of this effect is hypothesized

Therapy	Mode of action	Number of articles
CpG oligonucleotide (CpG ODN)	Is recognized by dendritic cells (DCs) and B cells. Activates T cells, natural killer (NK) cells, monocytes, neutrophils and plasma cell differentiation	8
Anti-cytotoxic T lymphocyte-associated protein 4 (anti CTLA-4)	Blocks the inhibitory receptor (CTLA-4) on the T cell and therefore activates the T cell for a specific immune response	6
Immature dendritic cells (DCs)	Phagocytosis of pathogens; antigen-presentation to other immune cells (among others T cells)	4
Natural killer (NK) cell therapy	Infusion with autologous NK cells to directly destroy tumour cells	4
Dendritic cell- cytokine induced killer (DC-CIK)	May act similarly to T cells or NK cells but is unrestricted to major histocompatibility complex	3
Granulocyte-macrophage colony-stimulating factor (GM-CSF)	A protein that functions as a cytokine and stimulates stem cells and can induce an immune cascade	3
Anti-programmed death-ligand 1 (PDL-1)	Blocks the receptor programmed death 1 on the tumour cell. This results in the activation of the T cell to induce a specific immune response	1

**Table 2:** Overview of the most studied factors of cryoablation and the immune system in the reviewed papers

Most studied factors of cryoablation and the immune system		
Mice		Total of 28 articles
Survival	↑	17/17
Rechallenge of primary tumour	↑	12/12
Reduction in distant metastasis	↑	10/10
Cytokine release	↑	16/17
IFN-y release	↑	16/17
TNFα release	↑	4/5
IL-4	↑	1/7
IL-10	↑	1/4
Th1/Th2 cytokine ratio	↑	10/0
CD4+ infiltration	↑	12/16
CD8+ infiltration	↑	14/19
Treg	↓	5/6
NK cells	↑	3/4
Human		Total of 17 articles
Survival	↑	8/8
Quality of Life	↑	4/4
Cytokine release	↑	6/7
IFN-y release	↑	6/7
TNFβ release	↑	3/4
IL-10	↓	3/5
IL-4	↓	3/4
IL-2	↑	3/5
Th1/Th2 cytokine ratio	↑	5/0
CD4+ infiltration	↑	5/8
CD8+ infiltration	↑	5/8
Treg	↓	2/2
NK cells	↑	4/4

### Breast Cancer

Cryoablation was approved for the treatment of fibroadenomas for over a decade [30]. In 2016, the American College of Surgeons Oncology Group (ACOSOG) alliance considered cryoablation as an effective treatment for unifocal ductal cancer, with a success rate of complete tumour ablation of 92% after correction for multifocal disease [31]. Also, for the treatment of stage IV breast cancer, cryoablation is a safe and effective procedure to control the disease and debulks the tumour in the breast [32].

Although antibodies are part of the treatment for human epidermal growth factor receptor 2 (HER2)-positive breast cancers, no active forms of immunotherapy such as immune checkpoint inhibitors are currently approved for breast cancer. Recently, the interim analysis of the Impassion 130 study with the combination of nab-paclitaxel plus atezolizumab revealed an impressive 10 months of improvement in overall survival compared with chemotherapy alone in PD-L1-positive triple negative metastatic breast cancers [33]. Other immune checkpoint blocking antibodies, such anti-CTLA-4 antibody, are also under investigation [34].

One of the first reports confirming the immunogenicity of cryoablation in breast cancer was in a mammary mouse model. After cryoablation or surgery, mice were re-challenged with tumour cells and only 16% of the cryoablated exhibited tumour development compared to 86% of the surgically treated mice [35].

In a metastatic breast cancer mouse model, cryoablated mice treated with a high freeze rate (100% cryoablation cycle) showed an improvement in overall survival with significant reduction in the number of pulmonary metastasis compared to treatment with a low freeze rate (10% cryoablation cycle) or those treated with surgery [19]. When cryoablation was combined with injection of CpG ODN (a single-strand DNA molecule that acts as a toll-like receptor (TLR) agonist to stimulate and mature DCs) in mice, less tumour recurrence and secondary tumour growth were seen after the re-challenge compared to the mice that received cryoablation alone or surgery [36]. No significant difference was reported between CpG ODN alone or in combination with cryoablation groups, leaving the added role of cryoablation to CpG ODN injection undetermined regarding cytokine release and potential immune activation [36].

Other studies have investigated the combination of cryoablation with an immune checkpoint inhibitor (ipilimumab) 7 days before mastectomy in a group of patients with early-stage breast cancer. The pilot study, including 19 patients, showed that this approach was a safe option without delaying the mastectomy [37]. A post hoc analysis was performed to assess the possibility of T cell receptor sequencing as a biomarker for T cell response to cryoablation, where no specific T cell response was observed [38]. Additionally, a phase II trial is ongoing where cryoablation in combined with ipilimumab and nivolumab before breast surgery in triple negative breast cancer patients after taxane-based neoadjuvant chemotherapy [39].

In another study, recurrent HER2-positive breast cancer patients were treated with the combination of cryoablation, trastuzumab and natural killer (NK) cell therapy (intravenous infusion of allogenic NK cells). These patients displayed a significantly prolonged progression-free survival (PFS), significantly larger numbers of T cells and Th1 cytokines together with a significantly reduction in the number of circulation tumour cells in the peripheral blood compared to patients only treated with cryoablation alone or cryoablation and NK cell therapy. To note, PFS was not reached in the triple combination group, and the significantly prolonged PFS could be due to trastuzumab [40]. Niu et al. evaluated the use of cryoablation in combination with immunotherapy of DC-CIK cells in metastatic breast cancer patients versus chemotherapy or cryoablation alone. The group of patients that received multiple cryoablations (several sites) in combination with immunotherapy displayed a significantly longer median overall survival compared to the other groups [41].

Together, cryoablation before mastectomy is feasible and its combination with immunotherapy, consisting of NK cell therapy, DC-CIK or anti-CTLA-4 antibody, is safe and effective in different stages in breast cancer. These results encourage further investigation into the combination of cryoablation and immunotherapy for breast cancer patients. Two trials are currently open which combine cryoablation with immune checkpoint inhibitors (Table 3) [42, 43].

**Table 3:** Overview of clinical trials of cryoablation and immunotherapy

Cancer type	Tumour stage	Study phase	Study design	Therapy	End points	End date	Centre	Identifier
Breast cancer	Early/resectable disease	Pilot	Open label single arm	Pre-operative ipilimumab + nivolumab + cryoablation	Safety: number of adverse events, no secondary end points.	June 2019	Memorial Sloan Kettering Cancer Center, USA	NCT02833233
	Resectable disease	II	Prospective randomized parallel interventional	Peri-operative cryoablation, ipilimumab nivolumab vs pre-operative care	Distant disease-free survival*	May 2021	Cedars-Sinai Medical Center, USA	NTC03546686
Renal cell carcinoma	Metastatic disease	I	Open label single arm	Tremelimumab +/− cryoablation before surgery	Objective response rate by irRC*	March 2021	MD Anderson, USA	NCT02626130
	Stage I	–	Prospective observational cohort	Immune response of cryoablation vs RFA vs LPN	Immune response: number of leucocytes in tissue samples post ablation.	June 2019	UC Irvine, USA	NCT03409224
Prostate cancer	Localized disease	Basic science	Open label single arm	Immune response profile after total cryotherapy, focal cryotherapy, SBRT and radical prostatectomy	Evaluate change in blood cytokine profile	Sept 2019	Winthrop University Hospital, USA	NCT03331367
	Metastatic disease	II	Open label single arm	Pembrolizumab and cryosurgery in combination with short term androgen ablation	Proportion of men with PSA < 0.6 ng/mL PD-1/PDL-1 expression	Nov 2018	Sidney Kimmel Comprehensive Cancer Center, USA	NCT02489357
	Stage I tm IIB	I	Prospective randomized open label clinical trial	GM-CSF after cryoablation	Change in B cell, T cells and PSA levels	Dec 2018	University of Colorado Cancer Center, USA	NCT02250014
	Castration resistant disease with positive lymph nodes	I	Open label single arm	Cryoablation plus intratumoural immature dendritic cells	Maximum tolerated dose*	April 2019	Haukeland University Hospital, Norwegian	NCT02423928
Lung cancer	Stage IV	II	Open label single arm	Core needle biopsy and cryoablation added to continued treatment with immune checkpoint inhibitor	Response by RECIST*	March 2025	Massachusetts General Hospital, USA	NCT03290677
Melanoma	Stage III tm IV cutaneous melanoma	I + II	Open label single arm	Dendritic cell therapy after cryosurgery in combination with pembrolizumab	Response by RECIST Clinical benefit*	Oct 2022	Mayo Clinic, USA	NCT03325101
	Stage IV, HLA-A2 + no curative disease	I	Open label single arm	Radiofrequency therapy + RFA/CA+ GM-CSF injection	Level of immune response by heat shock protein and lymphocyte Response by RECIST*	May 2018	Mayo Clinic, USA	NCT00568763
Other	Metastatic Colorectal cancer	I/IIb	Open label single arm	Combining cryoablation with intra-lesional immunotherapy with AlloStim® with dose escalation	Safety of increased frequency of dosing* Tumour response RECIST and histopathology HRQoL	Feb 2018	MD Anderson Medical Center, USA	NCT02380443 NB this study was first designed in BC
	Palliative stetting of HCC or BTC	I/II	Open label single arm	Tremelimumab and durvalumab + RFA/CA/TACE	PFS*	Apr 2021	National institutes of health clinical centre, USA	NCT02821754
	Palliative stetting of HCC or BTC stage B and C	I	Clinical prospective non-randomised	Tremelimumab + RFA/CA/SBRT/TACE	Response Rate Time to tumour progression Overall survival*	Dec 2018	National Institutes of Health Clinical Center, USA	NCT01853618
Other	Non-Hodgkin lymphoma	I/II	Open label single arm	Intratumoral DC therapy after cryosurgery and pembrolizumab	Maximum tolerated dose* Complete responses Disease free survival rate Duration of response OS, PFS, HRQoL	Feb 2021	Mayo Clinic, USA	NCT03035331
	1. Oesophageal cancer, 2. Tongue cancer, 3. Ovarian cancer, 4. Laryngeal cancer, 5. Pharyngeal cancer, 6. Cervical cancer 7. Tumours in transplanted livers 8. Sarcoma	I/II	Open label single arm trial	Cryoablation and NK cell immunotherapy	Response, PFS, OS by RECIST*	Jul 2019	Cancer Institute in Fuda Cancer Hospital, China	NCT02843581 NCT02849379 NCT02849353 NCT02849314 NCT02849327 NCT02849340 NCT02849015 NCT02849366
	1. Breast cancer, 2. Liver cancer, 3. Lung cancer, 4. Gastric cancer, 5. Colorectal cancer, 6. Pancreatic cancer 7. Kidney cancer	II	Prospective randomized triple arm study	Activated CIK cells + anti-bispecific antibody with or without cryoablation vs conventional therapy	Objective response rate PFS TTP*	April 2021	Fuda Cancer Hospital, China	NTC03524261 NTC03484962 NTC03501056 NTC03554395 NTC03524274 NTC03509298 NTC03540199

*RECIST* response evaluation criteria in solid tumours, *irRC* immune-related response criteria, *PFS* progression-free survival, *OS* overall survival, *CR* complete response, *PR* partial response, *HRQoL* health-related quality of life, *DSS* disease specific survival, *LHRH agonist* luteinizing-hormone releasing-hormone agonist, *TTP* time tumour progression, *RFA* radiofrequency ablation, *CA* cryoablation, *SBRT* stereotactic body radiotherapy, *DC* dendritic cell, *CIK* cytokine-induced killer cells, *NK* natural killer, *TACE* transarterial chemo-embolisation, *GM-CSF* granulocyte-macrophage colony-stimulating factor, *LPN* laparoscopic partial nephrectomy, *PSA* prostate specific antigen, *PD-1* programmed cell death protein, *PDL-1* programmed cell death ligand

*Safety has been performed in all studies by number of adverse events

### Renal cell carcinoma

In RCC, cryoablation is most frequently used to treat stage I cancer (ideally smaller than 4 cm taken as the largest diameter) in patients not eligible for surgical resection [44, 45]. With optimal patient selection, results similar to partial nephrectomy can be achieved [46].

Immunotherapy for RCC has been used for quite some time, and nivolumab, a PD-1 inhibitor, is already approved for the treatment of RCC [47, 48].

Two animal studies showed the favourable effect of cryoablation in the microenvironment of RCC and in the kidney. The first study used two mice models, one with and one without injected RCC to observe an inflammatory immune response after cryoablation in the tumour or healthy kidney tissue. An infiltration of neutrophils, macrophages and CD4+ and CD8+ T cells was reported after cryoablation whereby no difference was observed after cryoablation of normal kidney tissue or tumour tissue [49]. Another study compared cryoablation with surgery and showed decreased tumour growth after the re-challenge of the tumour cells with significantly more T cells in the peripheral blood after cryoablation [50].

Kato et al. showed that in half of the patients with T1 RCC, a significant increase in T cell receptor (TCR) B CD3 clonotypes of T-cells in post ablation tissue and blood was seen with a low diversity (TCR clones were not evenly distributed anymore) [51]. In another clinical study, two sessions of cryoablation of the pulmonary metastases, each combined with two Intratumoural injections of granulocyte-macrophage colony-stimulating factor (GM-CSF), resulted in higher levels of NK cells, Th1 cytokines and T and B cells in the peripheral blood compared to baseline [52]. Lin et al. showed similar effects of allogeneic NK cell immunotherapy combined with cryoablation in 60 advanced RCC patients, and this treatment combination resulted in more tumour responses and decrease in Hounsfield units count than cryoablation on its own [53].

To summarise, cryoablation of RCC elicits an immune response and can be safely combined with GM-CSF and NK cell therapy. Currently, one trial is ongoing investigating the synergy of cryoablation with anti-PD-1 therapy (tremelimumab), and another trial investigates the effect of ablation of the immune system [54, 55].

### Prostate cancer

Cryoablation is currently being used to treat stage I prostate cancer. Cryoablation could also be considered as salvage treatment for local recurrence after radiation therapy. Future perspectives in prostate cancer shift towards a more targeted therapy where cryoablation may have an important role in prostate cancer [56].

Presently in prostate cancer, the only approved immunotherapy is sipuleucel-T (Provenge), a DC-based immunotherapy that sensitises dendritic cells with prostate antigens and is used as a therapeutic vaccine [57]. Other immunotherapies evaluated have failed to show improvement in overall survival [58–60]. Current developments are focusing on immunotherapy for the subgroup with defects in DNA-repair mechanisms, which include microsatellite instability and breast cancer gene mutations.

Waitz et al. reported a regression in secondary tumour growth with infiltration of CD4+ and CD8+ T cells and lower counts of Tregs in mice treated by cryoablation and anti-CTLA-4 antibodies [29]. Another study reported that the combination of cryoablation with anti-CTLA-4 antibodies reduced distant metastasis in mice together with a reduction in the number of Tregs; these were lowest on day 14 but returned to normal levels at day 21 [61]. Recently, combination therapy of androgen deprivation plus anti-PD-1, anti-CLTA-4 or placebo with or without cryoablation demonstrated a delay in distant tumour growth and decreased mortality in mice in the trimodal therapy groups [62].

Pre cryoablation tissue in high-risk Localised prostate cancer patients showed elevated numbers of Tregs compared to healthy volunteers. Numbers of Tregs decreased significantly after cryoablation in the prostate patients, and, conversely, 7 of 12 patients had an increase of suppressive function of Tregs measured by immunosuppressive assay of CD4^+^CD25^+^CD127^−^ which was linked to the probability of recurrence of the cancer in 2 patients [63]. Another clinical study reported significantly higher cytokines (TNFα and IFN-y) levels, increased T cell response to autologous tumour tissue (IFN-y ELISPOT assay) after 4 weeks and higher cytotoxic activity of T cells after 4 and 8 weeks (measured by luciferase assay) after cryoablation in 20 high-risk prostate patients [64]. In patients with metastatic hormone refractory prostate cancer, a combination of cryoablation and GM-CSF showed a 70% decrease of PSA levels and a median time to progression of 18 months. No correlation was seen between the increase tumour-specific T cell responses in the peripheral blood and the increased cytolytic activity (measured by luciferase assay) after 4 and 8 weeks [65]. The addition of cryoablation to androgen deprivation therapy (ADT) in 30 prostate cancer patients with bone metastases significantly improved progression-free, cancer-specific and overall survival compared to 30 prostate patients only treated with ADT [66].

No clinical trials have been performed so far to evaluate a combination of anti-CTLA-4 antibodies with cryoablation therapy in humans. Only immunotherapy with GM-CSF has been investigated. Currently, a phase II trial of the combination of pembrolizumab and cryosurgery in stage IV prostate patients is ongoing. In addition, other trials are ongoing searching for the relation between cryoablation and the effect on the immune system [67–70].

### Lung cancer

Percutaneous local ablative therapies are considered viable options for the treatment of stage IA non-small-cell lung carcinoma (NSCLC). Recurrent lesions after radiation therapy or surgery and metastatic lesions can be treated by means of ablation as well [71, 72].

Cryoablation of lung lesions is associated with lower pain levels and fewer complications in tumours located close to the chest wall and mediastinum or central lesions close to the hilum; however, no clinical randomised studies have been executed comparing the different percutaneous ablative therapies [73, 74].

NSCLC is a heterogeneous group of cancers which is known for high numbers of tumour-specific mutations that are linked with response to immunotherapy [75]. In recent years, several immunotherapies have been approved for the treatment of lung cancer, namely PD-1 checkpoint inhibitors nivolumab and pembrolizumab and the anti-CTLA-4 inhibitor ipilimumab.

Preclinical work in a mouse model revealed that Intratumoural injection of DCs with cryoablation elicits a Th1 response with higher levels of IFN-y and effector memory CD8+ T cells observed from spleen cells resulting in protection against secondary tumours and prolonged survival [76]. In another study, the addition of CpG ODN to the cryoablation plus DCs resulted in a significant reduction of new tumour growth, fewer metastasis development and a prolonged survival compared to all the therapies alone. A decrease in Tregs and increase in cytotoxic T cells were observed and linked to the better response in the combination group [77]. In another study, the same treatment combination showed a higher elevation of CD4+ and CD8+ T cells and IL-12, IFN-y and TNFα together with a delay in tumour growth and improved survival in mice treated with the combination therapy [78]. Takahashi et al. reported the greatest immune response (higher numbers of specific T cells and higher levels of stimulating cytokines) and the slowest tumour growth after two cycles of cryoablation compared to one or three cycles of cryoablation [79].

Clinical work combining cryoablation and allogenic intravenous NK cells showed an improvement in the quality of life and tumour response rates compared to cryoablation alone. Additional phase II/III trials must be conducted to reveal the potential benefits in larger patient groups before combination treatment is considered an alternative [80]. Another strategy, consisting of a combination of cryoablation with gefitinib, an inhibitor of epidermal growth factor receptor’s (EGFR) tyrosine kinase domain, showed significant improvement of overall response with a higher 1-year survival rate in patients treated with gefitinib and cryoablation compared to gefitinib alone [81]. Lastly, 166 metastatic NSCLC patients received either cryoablation alone, cryoablation followed by immunotherapy (DC-CIK) or chemotherapy or all three therapies. The survival of patients treated with cryoablation combined with chemo or immunotherapy was longer than treatment consisting of chemo or immunotherapy alone (18 and 17 months vs 8.5 and 12 months). The overall survival in patients that received the triple combination therapy (cryoablation, immunotherapy and chemotherapy) was significantly longer (27 months) compared to other groups [82].

These studies show positive results for the combination of cryoablation and stimulants to the immune system, NK cell therapy or DC-CIK, with improvement in survival. Clinical results are expected from a phase II study where cryoablation is combined with an immune checkpoint inhibitor [83, 84].

### Melanoma

Cryoablation is used for the treatment of benign superficial lesions, such as actinic keratosis, but it is not indicated as a treatment for primary melanoma. Only in unresectable lesions with high metastatic load, cryoablation may diminish tumour load by ablation of the primary site [85, 86]. The treatment of melanoma metastasis can be performed with cryoablation to slow down the rate of tumour spread [87, 88]. In metastatic mouse models, combinations with different immunostimulants (including TLR 9 and CPG) have shown an enhanced effect of cryoablation for suppression of new tumour growth [26, 89–91]. A pilot study observed the induction of endogenous heat-shock protein after administration of GM-CSF and radiofrequency ablation or cryoablation in metastatic melanoma patients, with a small number of subjects demonstrating the combination therapy as a feasible and safe therapeutic option [92]. The combination of cryoablation and immunotherapy may be beneficial in the metastatic stetting to overcome the limitations of the immunotherapy. Currently, two trials are open combining immunotherapy and cryoablation (Table 3).

## Conclusion

Cryoablation has proven to be successful for local control in various cancer types. Mostly, it is indicated for tumours at an early stage or those not eligible for surgery. The synergies of local ablative techniques together with systemic treatments are currently one of the most exciting developments of interventional oncology. Particularly, given that cryoablation provides a pool of antigens visible for the immune system to induce an immune-specific activation directed against the tumour cells. In this review, a total of 41 out of 45 publications show favourable effects for cryoablation when combined with other therapies, potentially enhancing the anti-cancer immune response. This emphasises the potential advantage of this ablation technique as an adjunct with immunotherapy.

RCC and NSCLC are the neoplasms where cryoablation and its synergy with immunotherapy are predominantly studied. Both, immunostimulants for the innate and adaptive immune system demonstrate feasibility and effectiveness. For the translation of the results seen in animals and small groups of patients, larger prospective trials need to be designed to first study safety and efficacy before moving into randomized controlled settings. In the next 5 years, several pilot, phase I and early II clinical trials currently in place will demonstrate whether or not the combination of cryoablation and the immune system will have a true beneficial effect. This benefit can come from two different approaches that is first the contribution of cryoablation to immunological systemic effect of immunotherapy. Second, the impact of immunotherapy to overcome the limitations of cryoablation related to the upregulation of PD-L1. The features of imaging and molecular biomarkers, such as PD-L1 and others will help to optimise the combination strategies.

Furthermore, the strategy of combining cryoablation with new therapeutic options (specifically DC vaccine, CAR T cells, oncolytic viruses and adoptive T cell transfer) will provide a broader scale of immune-boosting therapies that will hopefully translate into the clinics and may thereby provide even better outcomes when combined with cryoablation.

## Abbreviations

ACOSOG: American College of Surgeons Oncology Group
ADT: Androgen deprivation therapy
anti-CTLA-4: Anti-cytotoxic T lymphocyte-associated protein 4
CpG ODN: CpG oligodeoxynucleotide
CT: Computed tomography
DC: Dendritic cell
DC-CIK: Dendritic cell cytokine-induced killer
GM-CSF: Granulocyte-macrophage colony-stimulating factor
HSP: Heat shock protein
IL: Interleukin
NK: Natural killer
NSCLC: Non-small cell lung carcinoma
PD-1: Programmed cell death protein 1
PD-L1: Programmed cell death ligand 1
PFS: Progression-free survival
RCC: Renal cell carcinoma
TCR: T cell receptor
TLR: Toll-like receptor
TNFα: Tumour necrosis factor alpha
Treg: Regulatory T cell
